# Evaluation of Bt Corn with Pyramided Genes on Efficacy and Insect Resistance Management for the Asian Corn Borer in China

**DOI:** 10.1371/journal.pone.0168442

**Published:** 2016-12-22

**Authors:** Fan Jiang, Tiantao Zhang, Shuxiong Bai, Zhenying Wang, Kanglai He

**Affiliations:** State Key Laboratory for Biology of Plant Disease and Insect Pests, Institute of Plant Protection, Chinese Academy of Agricultural Sciences, Beijing, P. R. China; University of Tennessee, UNITED STATES

## Abstract

A Bt corn hybrid (AcIe) with two Bt genes (*cry1Ie* and *cry1Ac*) was derived by breeding stack from line expressing Cry1Ie and a line expressing Cry1Ac. Efficacy of this pyramided Bt corn hybrid against the Asian corn borer (ACB), *Ostrinia furnacalis*, was evaluated. We conducted laboratory bioassays using susceptible and resistant ACB strains fed on artificial diet or fresh plant tissues. We also conducted field trials with artificial infestations of ACB neonates at the V6 and silk stages. The toxin-diet bioassay data indicated that mixtures of Cry1Ac and Cry1Ie proteins had synergistic insecticidal efficacy. The plant tissue bioassay data indicated that Bt corn hybrids expressing either a single toxin (Cry1Ac or Cry1Ie) or two toxins had high efficacy against susceptible ACB. Damage ratings in the field trials indicated that the Bt corn hybrids could effectively protect against 1^st^ and the 2^nd^ generation ACB in China. The hybrid line with two Bt genes showed a higher efficacy against ACB larvae resistant to Cry1Ac or CryIe than the hybrid containing one Bt gene, and the two gene hybrid would have increased potential for managing or delaying the evolution of ACB resistance to Bt corn plants.

## Introduction

Corn (*Zea mays* L.) is not only a staple food crop, but also an important raw material for feed and industry. The Asian corn borer (ACB), *Ostrinia furnacalis* (Guenée) (Lepidoptera: Crambidae) can cause serious damage to corn production and quality. Despite consistent losses estimated at 6 to 9 million tons in an ordinary year in China, farmers do not effectively manage this pest due to the difficulties and cost of insecticide application, environmental concerns, and uncertainty about the efficacy of the management strategies [1].

The commercialization of genetically modified Bt crops provides an effective method for the management of some insect pests. However, wherever a large area of a Bt crop has been planted, sustainability is threatened by development of resistance in the target pests. A number of resistant insect strains have been selected under laboratory conditions, including the ACB [2] and the European corn borer, *O*. *nubilalis* [3–7]. Studies have indicated that field populations of *Plutella xylostella* [8] and glasshouse populations of *Trichoplusia ni* [9] evolved resistance to Bt spray formulations after their repeated use in pest management programs. The evolution of resistance has been documented in field populations of *Busseola fusca* targeted by Bt corn expressing Cry1Ab [10], *Spodoptera frugiperda* targeted by Bt corn expressing Cry1F [11] and *Helicoverpa zea* targeted by Bt cotton expressing Cry1Ac [12]. These studies demonstrate the potential for the evolution of resistance to Bt crops in target insects.

Insect resistance management (IRM) strategies, such as the high-dose/refuge strategy, have been used widely in developed countries [13–16], especially during the first decade when the crops expressed only a single Bt gene [17]. However, this strategy is not feasible on small farm holdings in China, particularly where corn is considered a natural refuge crop for Bt cotton [18]. More recently, gene pyramiding has been introduced for IRM. Pyramiding two or more Cry toxins with different target sites has been increasingly deployed [19]. Chang et al. [20] reported that Bt corn that contained the fused genes *cry1Ab/cry2Aj* or *cry1Ab/vip3DA* could protect corn from attack by a Cry1Ab-resistant strain of ACB. Zhao et al. [21] used the Bt crucifer-diamondback moth system to demonstrate that plants expressing pyramided Bt genes delayed the rate at which resistance evolved with the pest population. The success of this strategy is based on the assumption that cross-resistance between the different toxins does not occur [22]. Our previous studies showed no cross-resistance between Cry1Ie and Cry1Ac proteins in ACB [23].

In this study, field tests and bioassays were used to evaluate the effectiveness of a breeding stack of Bt corn expressing Cry1Ac and Cry1Ie against the ACB, and evaluate the synergism between Cry1Ac and Cry1Ie proteins.

## Materials and Methods

### Plant material

Two Bt corn lines, one with a single Bt gene expressing Cry1Ie toxin (Z31Ie) developed by the Institute of Crop Sciences, Chinese Academy of Agricultural Sciences, and the other expressing Cry1Ac toxin (Zh58Ac) developed by China Agriculture University, were crossed to develop a hybrid (AcIe) with two pyramided Bt genes. The non-transgenic counterpart lines (not near-isoline) Zong 31 (Z31) and Zheng 58 (Zh58) were used to develop hybrids Zh58Z31 as a negative control. Crosses between Z31Ie and Zh58 and/or between Zh58Ac and Z31 yielded the hybrids Zh58Ie and Z31Ac that only expressed Cry1Ie and Cry1Ac, respectively. Expression of Cry1Ac was 292.13 ng/g in tassels, 246.08 ng/g in whorl leaves, 79.51 ng/g in silks, and 233.76 ng/g in husks (unpublished data). An antibody for Cry1Ie was unavailable and its expression level was not determined. These hybrids were grown on the farm at the experimental station of the Institute of Plant Protection, Chinese Academy of Agricultural Science in Langfang, Hebei Province, China in 2013 and 2014. The planting dates were 21 May (spring corn) and 10 June (summer corn) each year. All plants were managed by following local cultural practices but no insecticide was applied.

### Insects

A susceptible strain (ACB-BtS) and Cry1Ac-, Cry1Ie-, and Cry1F-resistant strains (ACB-AcR, ACB-IeR, and ACB-FR, respectively) of ACB were used in this study. The ACB-BtS strain was obtained from a laboratory colony that was established from annual field collections in Hebei province and maintained on a semi-artificial diet [1]. The ACB-AcR strain was previously characterized and was selected from a field population collected from Shaan’xi, China in 2000 using trypsin-activated Cry1Ac toxin [24], and this strain had greater than a 1000-fold resistance to the Cry1Ac toxin [25]. Strains ACB-IeR and ACB-FR were selected for 23–24 generations with a final selection dosage at 4 μg/g of Cry1Ie toxin/diet or 25 μg/g of Cry1F toxin/diet. The LC_50_ of the ACB-IeR strain is greater than 940 μg/g and the resistance ratio (RR) is greater than 850 (unpublished data). The LC_50_ of the ACB-FR strain is greater than 1000, and the RR is higher than 1754 [26]. All of the ACB strains were reared at 27–28°C and 80% relative humidity (RH) under a photoperiod of 16:8 h (L: D). Neonates that hatched within 12 h of one another were used for bioassays and field infestations.

### Bt toxins for bioassays

The Cry1Ac protein was produced using a recombinant *Bacillus thuringiensis* (*Bt*) strain [27]. TheCry1Ie toxin was expressed as a recombinant protein in *Escherichia coli* [28]. Activated Bt toxins were produced by treating the protoxin with trypsin [29]. The susceptibility of the ACB-BtS, ACB-AcR and ACB-IeR strains to either a single Bt toxin protein (Cry1Ac or Cry1Ie) or a mixture of both Cry1Ac and Cry1Ie was determined by exposing neonates to a diet with a series of toxin concentrations of the Cry1Ac and Cry1Ie toxins. The ratios for combinations of the two toxins (Cry1Ac:Cry1Ie) were 0.17:0.83, 0.33:0.67, 0.50:0.50, 0.67:0.33, 0.83:0.17, 1:0, 0:1 (Table 1). Dose-response was assayed using the diet incorporated bioassay method as described by He et al. [30]. In the dose-response experiments, 9–13 concentrations of each combination of toxins plus a buffer control were employed. All of the bioassays were performed in duplicate on three different dates. The assays were conducted at 28±2°C, 80% RH and with a photoperiod of 16:8 h (L:D) for 7 days for scoring.

**Table 1:** Synergistic effects between Cry1Ac and Cry1Ie toxins on ACB.

ACB-strain	Toxins proportions (Cry1Ac:Cry1Ie)	LC_50_ (95%FL) (μg/g)	Expected LC_50_ (μg/g)	SF	χ^2^
ACB-BtS	0.17:0.83	6.13 (4.20–8.34)	13.93	2.3	66.747
	0.33:0.67	2.85 (1.92–3.79)	8.83	3.1	42.951
	0.50:0.50	1.43 (0.97–1.96)	6.46	4.5	47.116
	0.67:0.33	2.28 (1.88–2.71)	5.09	2.2	36.012
	0.83:0.17	0.80 (0.65–0.97)	4.21	5.3	40.859
	1:0	3.58 (3.11–4.08)	–	–	24.247
	0:1	32.97 (24.03–43.43)	–	–	47.467
ACB-AcR	0.17:0.83	86.27 (72.52–100.05)	59.88	0.6N	14.457
	0.33:0.67	63.73 (52.32–75.38)	73.53	1.2N	23.621
	0.50:0.50	74.90 (52.07–100.00)	96.15	1.3N	48.133
	0.67:0.33	32.74 (23.09–42.20)	136.99	4.2	28.667
	0.83:0.17	42.50 (34.39–50.36)	243.90	5.7	8.529
	1:0	>1000	–	–	–
	0:1	50.2 9(40.47–61.08)	–	–	41.664
ACB-IeR	0.17:0.83	19.09 (15.530–23.11)	37.59	2.0	34.692
	0.33:0.67	5.47 (4.15–7.06)	20.83	3.8	49.292
	0.50:0.50	5.83 (4.27–7.62)	14.29	2.5	53.200
	0.67:0.33	5.36 (3.09–7.68)	10.99	2.1	47.706
	0.83:0.17	3.24 (2.37–4.31)	8.85	2.7	42.408
	1:0	7.43 (6.15–8.71)	–	–	22.438
	0:1	>200	–	–	–
ACB-FR	0.17:0.83	25.55 (21.81–29.53)	42.92	1.7	29.077
	0.33:0.67	21.48 (18.70–24.35)	32.36	1.5	12.321
	0.50:0.50	14.19 (11.43–17.16)	25.97	1.8	38.739
	0.67:0.33	4.83 (4.00–5.76)	21.74	4.5	45.239
	0.83:0.17	9.14 (7.92–10.43)	18.66	2.0	15.086
	1:0	16.35 (12.35–20.42)	–	–	42.338
	0:1	63.52 (49.34–78.29)	–	–	43.428

SF: synergism factor;

N: no synergism effect

The 50% lethal concentration (LC_50_) with 95% fiducial limits (FL) was determined via a Probit analysis with PoLoPlus V 1.0 (LeOra Software Company, Petaluma, USA) and the concentration was expressed as μg toxin per gram of artificial diet. The expected LC_50_ value and the synergistic factor (SF) for the protein combinations were calculated using the formula of Tabashnik et al. [31], i.e. the expected LC_50_ without synergism of a mixture of *x* and *y* proteins was calculated as

$${\text{LC}}_{50\text{(}e\text{)}}={\left[\frac{r_{a}}{{\text{LC}}_{\text{50(}x\text{)}}}+\frac{r_{b}}{{\text{LC}}_{\text{50(}y\text{)}}}\right]}^{-1}.$$

where LC_50(*x*)_, LC_50(*y*)_ and LC_50(*e*)_ are, respectively, the LC_50s_ of proteins *x* and *y* and the expected LC_50_ without synergism of the mixture of the two proteins. For the mixture of *x* and *y* proteins, if the LC_50(*e*)_ was greater than the higher limit of the 95% confidence interval for the observed LC_50(m)_, synergism was indicated between the two proteins. SF = LC_50(*e*)_/LC_50(*m*)_.

### Efficacy of plant tissue bioassays

The efficacy of the three types of Bt corn or hybrids expressing either one (*cry1Ac* or *cry1Ie*) or both genes was evaluated against the four ACB strains (ACB-BtS, ACB-AcR, ACB-FR, and ACB-IeR). The whorl leaves, immature tassels, fresh silks and husks were tested using the method described by Xu et al. [2]. The non-extension whorl leaves were cut into small sections (approximately 1 cm length and 1 cm width) and placed in each well of 24-well trays. One neonate was placed individually into each well containing one leaf cut that was replaced with fresh tissue every other day. To maintain a suitable moisture level inside the well, each tray was covered with a piece of moistened filter paper and a lid. Fresh silks and husks were assayed using plastic dishes (7 cm high × 9 cm diameter). Each container with a corn silk or husk sample was infested with 20 neonates from each of the four ACB strains. The numbers of survivors was recorded after 2, 4, and 6 days. Each test was replicated six times. All plant tissue bioassays were conducted in a growth chamber at 70–80% RH, 28–30°C, and with a photoperiod of 16:8 h (L:D). Larval mortality was compared using one-way ANOVA with PROC MIXED (SAS Institute 2004). Mortality values were transformed using the arcsine (x^0.5^) to normalize the treatment variance [32]. The mean mortality was compared using Fisher’s Protected LSD test.

### Field evaluations

The efficacy of different Bt corn events or hybrids on the 1^st^ and 2^nd^ generation ACB was evaluated in the field with an artificial infestation method as in He et al. [33]. A randomized complete block design was used for planting the hybrids in the field efficacy trials. Each hybrid was replicated six times in 2013 and three times in 2014. Each treatment plot contained two 5 m rows (approximately 40 plants). The 1^st^-generation infestation was conducted at the V6 whorl stage with 40–60 ACB-BtS neonates per plant [33]. The infestation was conducted in the late afternoon. The leaf-feeding rates was estimated on a scale of 1 to 9 (1 = the most resistant and 9 = the most susceptible [33]) 14 days after infestation. Stalk damage was evaluated by dissecting the plants to record the number and length of the stalk tunnels. The 2^nd^-generation infestation was conducted at the silking stage (R1) with 40–60 ACB-BtS neonates per silk mass. Damage ratings were estimated during the harvest by dissecting the stalks and cobs to record the number and length of the tunnels. The efficacy of ACB-BtS was compared using a one-way ANOVA conducted via the PROC MIXED routine (SAS Institute 2004), and the mean values were separated with Fisher’s Protected LSD test. The analyses included damage ratings, the number of larvae in the stalks and cobs, and the number and length of the tunnels in the stalks and cobs. The leaf-feeding ratings were evaluated for all plants infested in each plot and used to calculate a single mean rating.

## Results

### Synergism between Cry1Ac and Cry1Ie in diet assays

For the ACB-BtS strain, the observed LC_50_ for each of the five combinations of the Cry1Ac and Cry1Ie toxins was less than 1/5 to 1/2 of the LC_50_ expected in the absence of synergism (Table 1), suggesting a 2- to 5-fold synergism between the Cry1Ac and Cry1Ie toxins. For ACB-AcR strain, the expected LC_50_ values of the combinations (Cry1Ac:Cry1Ie at 0.17:0.83, 0.33:0.67 and 0.5:0.5) were within the 95%FL for the observed LC_50_ values, suggesting that no synergism had occurred. In contrast, the observed LC_50_ values of combinations 0.67:0.33 and 0.83:0.17 were less than 1/4 and 1/5 of the expected LC_50_ in the absence of synergism, respectively (Table 1), suggesting that 4- and 5-fold synergisms had occurred. For the ACB-IeR and ACB-FR strains, comparisons between the observed and expected LC_50_ values showed a 1.5–4.5-fold synergism of the two toxins. These data suggested that a combination of the Cry1Ac and Cry1Ie toxins was more effective against the ACB strains than a single toxin and that there was no cross-resistance between the Cry1Ac and Cry1Ie toxins in ACB.

### Efficacy of plant tissues

All Bt corn events or hybrids had high efficacy against ACB-BtS larvae with >95% mortality after two days when the larvae were fed on fresh whorl leaves, immature tassels and husks of the Bt corn hybrids (Table 2). In contrast, most larvae developed normally when they fed on the same non-Bt corn hybrids. No significant difference in mortality among the three Bt corn hybrids (97.9–100%) was evident after four days, with significantly higher mortality than on the non-Bt corn hybrid. The efficacy of fresh silk of the Bt corn hybrids was lower than the efficacy of other tissues against ACB-BtS larvae, with 64.2–85.0% after two days and 69.2–93.3% after four days.

**Table 2:** Mortality of ACB larvae of four strains fed on plant tissues of Bt corn and non-Bt corn hybrids.

Insect strains	Corn hybrids	Mortalities %											
		Whorl			Tassel			Silk			Husk		
		2 d	4 d	6 d	2 d	4 d	6 d	2 d	4 d	6 d	2 d	4 d	6 d
ACB-BtS	Zh58Z31	6.9±1.4 c	7.6±1.3 b		11.8±1.3 b	13.9±1.4 b		10.8±2.0 c	15.8±2.7 c	27.5±3.6 c	9.2±2.0 c	15.8±3.3 b	19.2±3.3 b
	Zh58Ie	99.3±0.7 a	100.0±0.0 a		98.6±1.0 a	100.0±0.0 a		64.6±2.7 b	69.2±3.1 b	78.8±1.1 b	95.8±1.4 b	99.2±0.5 a	99.6±0.4 a
	Z31Ac	95.1±2.0 b	97.9±1.4 a		99.3±0.7 a	100.0±0.0 a		85.0±5.32 a	93.3±4.9 a	95.8±3.3 a	100.0±0.0 a	100.0±0.0 a	100.0±0.0 a
	AcIe	96.5±1.3 ab	99.3±0.7 a		100.0±0.0 a	100.0±0.0 a		64.2±6.0 b	80.0±2.9 b	82.5±2.5 b	96.7±3.3 ab	98.3±1.7 a	99.2±0.8 a
	*F*_3, 20_	176.55	339.6		402.5	3220.74		39.92	50.75	61.97	142.42	207.79	277.11
	*P*	<.0001	<.0001		<.0001	<.0001		<.0001	<.0001	<.0001	<.0001	<.0001	<.0001
ACB-AcR	Zh58Z31	18.1±1.4 c	27.8±1.8 c	31.3±1.4 c	13.9±1.8 c	17.4±2.0 c	21.5±3.3 c	5.8±3.3 c	10.0±3.7 c	21.7±4.8 b	5.8±1.5 d	10.8±2.4 d	16.7±2.8 d
	Zh58Ie	98.6±0.7 a	100.0±0.0 a	100.0±0.0 a	97.2±1.0 a	97.6±1.1 a	98.6±0.7 a	32.1±3.3 a	42.1±4.3 a	54.2±3.4 a	76.7±4.1 b	91.7±1.4 b	94.2±1.4 b
	Z31Ac	36.1±3.3 b	52.1±2.8 b	62.5±1.9 b	45.8±2.9 b	54.9±2.0 b	60.4±3.2 b	16.7±2.1 b	19.2±2.4 b	38.3±2.1 a	33.3±5.6 c	40.0±7.3 c	50.0±8.4 c
	AcIe	99.3±0.7 a	100.0±0.0 a	100.0±0.0 a	95.8±1.5 a	96.5±1.7 a	97.9±1.4 a	18.3±3.8 b	23.3±5.1 b	53.3±6.2 a	96.7±2.1 a	98.3±1.1 a	100.0±0.0 a
	*F*_3, 20_	339.60	923.53	1575.35	165.55	139.27	138.52	10.81	9.19	9.07	91.03	112.65	111.9
	*P*	<.0001	<.0001	<.0001	<.0001	<.0001	<.0001	0.0002	0.0005	0.0005	<.0001	<.0001	<.0001
ACB-IeR	Zh58Z31	6.9±1.4 d	13.9±2.3 c	16.7±1.9 c	6.3±1.8 c	13.2±2.0 c	17.4±2.3 c	3.3±1.7 c	13.3±1.1 b	20.0±1.84 c	5.8±1.5 c	13.3±1.1 c	20.8±1.5 c
	Zh58Ie	22.9±3.1 c	26.7±2.7 b	36.5±4.4 b	46.5±1.3 b	51.7±1.3 b	55.6±1.0 b	10.8±1.9 b	20.8±1.7 b	27.5±2.7 b	33.3±2.5 b	38.3±5.2 b	49.6±4.7 b
	Z31Ac	95.8±1.1 b	96.5±1.3 a	98.6±0.9 a	97.2±1.4 a	97.2±1.9 a	99.3±0.7 a	20.0±3.2 b	31.7±3.8 a	47.5±2.5 a	97.5±1.7 a	99.2±0.8 a	100.0±0.0 a
	AcIe	99.3±0.7 a	100.0±0.0 a	100.0±0.0 a	97.2±1.4 a	99.3±0.7 a	100.0±0.0 a	31.7±3.8 a	36.7±4.4 a	42.5±4.0 a	100.0±0.0 a	100.0±0.0 a	100.0±0.0 a
	*F*_3, 20_	337.11	432.20	293.11	135.80	242.24	573.49	19.09	12.07	269.41	256.45	323.36	478.90
	*P*	<.0001	<.0001	<.0001	<.0001	<.0001	<.0001	<.0001	<.0001	<.0001	<.0001	<.0001	<.0001
ACB-FR	Zh58Z31	10.4±1.4 d	14.6±1.8 c	19.4±2.3 c	9.7±1.8 c	18.1±1.4 b		8.3±1.1 c	17.5±2.4 c	31.7±3.1 bc	11.7±1.1 d	19.2±3.0 c	32.5±2.8 c
	Zh58Ie	98.3±1.0 b	100.0±0.0 a	100.0±0.0 a	98.3±0.8 ab	100.0±0.0 a		31.3±2.8 a	33.8±3.3 ab	42.1±2.3 b	91.9±2.5 b	96.7±2.0 a	100.0±0.0 a
	Z31Ac	78.5±2.3 c	90.3±1.8 b	96.5±1.3 b	95.8±1.9 b	100.0±0.0 a		20.8±4.2 b	23.3±5.4 bc	27.5±5.9 c	60.4±5.7 c	66.7±4.9 b	76.7±4.6 b
	AcIe	100.0±0.0 a	100.0±0.0 a	100.0±0.0 a	99.3±0.7 a	100.0±0.0 a		40.8±4.7 a	44.2±5.1 a	62.5±3.1 a	99.2±0.8 a	100.0±0.0 a	100.0±0.0 a
	*F*_3, 20_	308.18	804.1	348.1	191.43	3717.97		17.47	7.52	13.68	123.28	130.31	211.86
	*P*	<.0001	<.0001	<.0001	<.0001	<.0001		<.0001	0.0015	0.0002	<.0001	<.0001	<.0001

The mean±SE in the same column followed by the same letter were not significantly different by Fisher’s Protect LSD test (*P* ≥ 0.05).

The Zh58Z31 corn line is a hybrid of the non-transgenic counterpart lines Zong 31 (Z31) and Zheng 58 (Zh58) was used as a negative control. The Zh58Ie corn line is a hybrid that only expresses Cry1Ie. The Z31Ac corn line is a hybrids that only expresses Cry1Ac. The AcIe corn strain, is a hybrid that expresses both Cry1Ac and Cry1Ie.

The mortality of the ACB-AcR larvae varied significantly among the three corn hybrids (Table 2). When larvae were infested on tissues of Z31Ac that expressed only the Cry1Ac toxin, most survived for 6 days; ACB larvae are resistant to Cry1A protein [25]. The AcIe and Zh58Ie hybrids that expressed Cry1Ie toxin had high efficacy; most larvae could not survive longer than 2- or 4- days. In contrast, most larvae developed normally when fed on tissues of the non-Bt corn hybrid. Similarly, larval mortality varied significantly among the three corn hybrids for the ACB-IeR strain (Table 2).

The corn hybrid Zh58Ie, which expressed only the Cry1Ie toxin had low efficacy against ACB-IeR larvae while the hybrids AcIe and Z31Ac, which expressed the Cry1Ac toxin, had a significantly higher efficacy than Zh58Ie after 4–6 days. The mortality of the ACB-FR larvae also varied significantly among the three corn hybrids (Table 2), showing a lower efficacy for hybrid Z31Ac than Zh58Ie and AcIe, especially after 2–4 days.

These results indicate that all three Bt corn events or hybrids had high efficacy against ACB-BtS. The AcIe hybrid that expressed both toxins (Cry1Ac and Cry1Ie) had high efficacy against ACB larvae of the susceptible strain or if resistant to one of the toxins, which also suggests no cross-resistance between the Cry1Ac and Cry1Ie toxins in ACB. However, the hybrids that expressed a single Bt toxin (Z31Ac or Zh58Ie) had low efficacy against ACB strains resistant to Cry1Ac or Cry1Ie, respectively.

### Efficacy in field tests

#### Whorl stage

The field tests of the artificial infestation of ACB-BtS at V6 stage showed minimal leaf feeding damage on Bt corn hybrids with either a single (*cry1Ac* or *cry1Ie*) gene or both Bt genes. The leaf feeding ratings were significantly lower in the three Bt hybrids than in the Zh58Z31 control (Table 3). The number of tunnels per plant was significantly higher in the non Bt hybrid than in the Bt corn hybrids. These results indicate that all three Bt corn hybrids provided significant protection against first generation ACB, but none at a level that can be considered high-dose.

**Table 3:** Damage ratings of corn lines infested with ACB at V6 whorl stage.

Hybrids	Leaf feeding ratings				Tunnels /100 Plants			
	2013		2014		2013		2014	
	Spring corn	Summer corn	Spring corn	Summer corn	Spring corn	Summer corn	Spring corn	Summer corn
Z**h**58Z31	7.5±0.2 a	5.8±0.2 a	6.6±0.3 a	6.7±0.0 a	78.9±8.2 a	98.9±5.7 a	35.8±4.8 a	108.0±7.8 a
Z**h**58Ie	1.4±0.1 b	1.4±0.1 bc	1.3±0.1 b	1.5±0.0 c	15.6±4.6 b	12.2±0.0 c	14.0±4.0 b	56.7±8.7 b
Z31Ac	1.4±0.2 b	1.7±0.1 b	1.5±0.0 b	1.9±0.1 b	18.9±3.8 b	39.4±5.2 b	8.9±1.1 b	37.0±15.3 b
AcIe	1.0±0.0 b	1.2±0.0 c	1.3±0.1 b	1.5±0.1 c	8.9±1.4 b	12.2±2.8 c	12.1±1.1 b	22.3±8.7 b
*F*	553.23	321.69	291.60	1299.84	40.52	92.56	14.54	12.62
df	3, 20	3, 20	3, 8	3, 8	3, 20	3, 20	3, 8	3, 8
*P*	<0.0001	<0.0001	<0.0001	<0.0001	<0.0001	<0.0001	0.0013	0.0021

The mean±SE in the same column followed by the same letter were not significantly different by Fisher’s Protect LSD test (*P* ≥ 0.05). The Zh58Z31 corn line is a hybrid of the non-transgenic counterpart lines Zong 31 (Z31) and Zheng 58 (Zh58) was used as a negative control. The Zh58Ie corn line is a hybrid that only expresses Cry1Ie. The Z31Ac corn line is a hybrids that only expresses Cry1Ac. The AcIe corn strain, is a hybrid that expresses both Cry1Ac and Cry1Ie.

#### Silking stage

The number of tunnels per 100 plants was significantly lower in the three Bt corn hybrids than in the non-Bt corn hybrid (Table 4). No significant difference was evident among three Bt hybrids either with one or two Bt genes. The results indicate that the three Bt corn hybrids provided significant protection in the field against second generation ACB, but none at a level that can be considered high-dose.

**Table 4:** Damages of corn lines infested with ACB at R1 silking stage.

Hybrids	Tunnels /100 Plants			
	2013		2014	
	Spring corn	Summer corn	Spring corn	Summer corn
Z**h**58Z31	56.7±3.9 a	92.8±7.0 a	133.8±19.8 a	106.7±21.1 a
Z**h**58Ie	5.6±2.8 b	5.0±1.4 b	32.1±7.3 b	37.8±7.5 b
Z31Ac	4.4±2.4 b	7.8±2.2 b	82.7±31.8 ab	22.0±4.5 b
AcIe	3.3±0.9 b	4.4±1.7 b	44.3±22.4 b	15.3±2.3 b
*F*	93.71	130.42	4.28	13.29
df	3, 20	3, 20	3, 8	3, 8
*P*	<0.0001	<0.0001	0.0445	0.0018

The mean±SE in the same column followed by the same letter were not significantly different by Fisher’s Protect LSD test (*P* ≥ 0.05). The Zh58Z31 corn line is a hybrid of the non-transgenic counterpart lines Zong 31 (Z31) and Zheng 58 (Zh58) was used as a negative control. The Zh58Ie corn line is a hybrid that only expresses Cry1Ie. The Z31Ac corn line is a hybrids that only expresses Cry1Ac. The AcIe corn strain, is a hybrid that expresses both Cry1Ac and Cry1Ie.

## Discussion

The laboratory bioassay results indicated that corn hybrids that expressed Bt gene(s) effectively reduced plant damage from ACB infestation. The hybrid that expressed two toxins (Cry1Ac and Cry1Ie) had high efficacy against susceptible ACB larvae or the larvae of a strain resistant to one toxin. The Cry1Ie toxin had high efficacy against ACB resistant to Cry1Ac. Our previous studies showed that, Cry1Ie had no cross resistance with Cry1Ac or Cry1F but Cry1F had low cross resistance with Cry1Ac [23]. Gene pyramiding is a good strategy for IRM and can retard the evolution of pest resistance in transgenic crops [8]. Planting corn hybrids with the *cry1Ac/cry1Ie* gene pyramid may prove beneficial to IRM of ACB because individuals resistant to a single toxin did not survive on the pyramided plants in the laboratory test.

Transgenic Bt corn affects ACB larvae via the ingestion of the Bt protein. Therefore, it is important to know the efficacy of the whorl leaf, tassel and silk tissues on which the neonate larvae initially feed. First generation neonates of ACB tend to eat whorl leaves [1]. At the end of first generation and the beginning of the second generation, eggs are deposited on corn plants during the late-whorl stage. Some of early instar larvae directly attack the immature tassels, and most second generation neonates attack the fresh silk. Different tissues of Bt maize have efficacy against pest [34], and our result indicated that whorl leaves, tassels, silks and husks are efficacious against ACB neonates.

The use of a combination of Bt toxins has become one of the most effective methods for increasing the efficacy of the Bt crop. Previous studies have demonstrated that synergism might occur between Cry and Cyt toxins [35, 36]. Xue et al. [37] reported Cry1Aa and Cry1C showed synergistic activity against *Spodoptera exigua* and *Helicoverpa armigera*. Avilla et al. [38] compared several Bt proteins and indicated Cry1Ac4, Cry2Aa1, Cry9Ca, Cry1Fa1, Cry1Ab3, Cry2Ab2, Cry1Da, and Cry1Ja significantly inhibited growth of *Helicoverpa armigera*. Other bioassay results showed synergistic or antagonistic effects of Bt toxins on insects [39–41]. Different hypotheses have been proposed to explain the molecular mechanism of synergism, including (i) the improvement of toxin docking and membrane insertion [42] and (ii) the destruction of the midgut peritrophic matrix, which increases toxin permeability [43, 44].

Our study revealed that a combination of Cry1Ac and Cry1Ie had synergistic toxicity against susceptible ACB strains or strains resistant to one of the proteins. An appropriate proportion of the toxins expressed in transgenic plants can maximize the efficacy and delay insect resistance development. The combinations of Cry1Ac and Cry1Ie can also be used as an IRM method for delaying the rate of resistance development of ACB to either or both Bt proteins.
